# Resilient Moderating Effect between Stress and Life Satisfaction of Mothers and Fathers with Children with Developmental Disorders Who Present Temporary or Permanent Needs

**DOI:** 10.3390/ejihpe14030032

**Published:** 2024-02-23

**Authors:** Óscar Gavín-Chocano, Inmaculada García-Martínez, Virginia Torres-Luque, Lara Checa-Domene

**Affiliations:** 1 Department of Pedagogy, University of Jaén, 23071 Jaén, Spain; ogavin@ujaen.es; 2 Department of Didactics and School Organization, Faculty of Education, University of Granada, Campus de Cartuja s/n, 18071 Granada, Spain; virginia_torres_luque@ainper-linares.org (V.T.-L.); laracd@correo.ugr.es (L.C.-D.); 3 C.A.I.T. AINPER—LINARES, 23700 Linares, Spain

**Keywords:** parental stress, resilient attribute, life satisfaction, developmental disorder

## Abstract

(1) Background: The stress experienced by parents with children with developmental disorders who present temporary or permanent needs, makes them experience a series of daily situations that may be linked to coping strategies. Resilient attributes are considered one of the factors that have a decisive influence on the behavior of parents related to raising their children and that affect greater well-being and life satisfaction. The objective of this research is to study the mediating effect of resilient attributes between parental stress and life satisfaction; (2) Methods: In this study, mothers and fathers of boys and girls from 0 to 6 years old with developmental disorders from different Early Childhood Care Centers (CAIT) in the Province of Jaén (Andalusia) [Spain]. Of them, 96 are mothers (78.0%) and 27 are fathers (22.0%), with a mean age of 37.85 years (±5.043). The *Parenting Stress Index-Short Form* (PSI-SF), *Resilience Scale* (RS-14), and *Satisfaction with Life Scale* (SWLS) were used. The structural equations model (PLS-SEM) was applied to estimate the proposed theoretical model, from an explanatory-predictive perspective; (3) Results: The results showed the coefficients of determination Parental distress [(Q^2^ = 0.144); (R^2^ = 0.329)]; Personal competence [(Q^2^ = 0.106); (R^2^ = 0.246)]; Acceptance of self and life [(Q^2^ = 0.094); (R^2^ = 0.172)] and Life satisfaction [(Q^2^ = 0.182); (R^2^ = 0.563)], in the estimation of the reflective model, indicating a moderate fit; (4) Conclusions: The present investigation is not conclusive; however, the implications of these findings are discussed and suggestions for future research are considered.

## 1. Introduction

The family is configured as the first socializing agent of any child, since its figure becomes essential for adequate cognitive, social, and economic stimulation, as well as for its comprehensive development [1,2]. In the case of children with educational needs, it is even more, being a great challenge for parents that in certain circumstances generates stress [3].

This fact has led to a growing interest in the scientific literature in recent years. Some research is focused on studying how stress, specifically parental stress, is linked in interaction with other variables, such as resilience and life satisfaction, that affect the confrontation of stressful and threatening situations of those families who have children with transitory or permanent needs [4].

Early intervention programs include children with “Deficits”, “Disabilities”, as well as those at high risk of developing disabilities. They are mostly integrated into primary prevention programs as part of a child protection strategy. This represents a change from the previous model which limited intervention for a specific number of individuals. This evolution has significantly improved the quality of early intervention services by establishing three levels of intervention in this discipline.

Nowadays, the group of children, who present transitory or permanent needs derived from developmental disorders, has variations. They are divided into three main categories. The first group includes children diagnosed with documented impairments or disabilities, such as motor, cognitive, language, sensory, behavioral, emotional, and other developmental disorders. The second group includes those children who, during their pre-, peri- or postnatal period, have faced situations that could affect their development, such as prematurity, low birth weight, or anoxia [5]. Finally, children at psychosocial risk are those who live in unfavorable conditions, such as lack of care, inadequate interactions, maltreatment, or neglect, which may impact their maturation [6].

In this context, bearing in mind the contributions of Abidin [7], the stress in the parent–child relationship is closely linked to those aspects related to upbringing, that is, to the parent’s reaction to a situation that is perceived as threatening or overwhelming of their resources and consequently jeopardizes their emotional well-being. In the review of literature, the bond between stress and the daily care of children shows the effect that they have on the psychological health of parents and relationships with their children, being even greater on boys and girls who present transitory or permanent needs [8,9].

There are factors that determine a higher level of parental stress such as the influence of the prognosis [10], the physical characteristics of the children [11] behavioral problems [12], and limitations in the social skills of children [13]. Previous studies have corroborated that high levels of parental stress are related to self-regulation and behavior problems in children [14] and negative parent–child relationships [15]. Mainly, there are two opposing perspectives that can be adopted. On the one hand, it is likely that parents, when confronted with their child’s difficulties, demonstrate an interaction characterized by excessive demands, which may lead children to be unable to respond adequately. This may result in feelings of inadequacy and failure. This dynamic results in frustration on the part of parents. On the other hand, some parents may choose to neglect or overprotect. Choosing not to put pressure on the child, those who take on this role avoid acknowledging the child’s difficulties. This may prevent children from fully developing their abilities [16].

However, these stressful situations and the way in which parents deal with the deficiencies of children with developmental disorders who present transitory or permanent needs can occur in a circumstantial and positive way, or negative, such as an attitude of avoidance and resistance [17]. According to *Libro Blanco de la Atención Temprana*, Early Care is defined as a set of actions aimed at boys and girls from 0 to 6 years old, as well as their families and environments, with the main aim of addressing temporary or permanent needs of those children who have developmental disorders or are at risk of suffering them [18]. Child development involves the progressive acquisition of fundamental functions such as postural control, manipulation, autonomous movement, communication, verbal language, and social interaction. This process is closely conditioned by genetic and environmental factors, which can be biological, psychological, or social. During the early childhood stage, the nervous system is characterized by its immaturity and plasticity. Therefore, it is crucial that Early Care interventions are carried out as soon as possible once a developmental problem is detected. These interventions, which must consider the global nature of these people, must be identified by a team of professionals with an interdisciplinary or transdisciplinary orientation.

Different investigations have related stress with resilient attributes, as an adaptive response and a measure of protection when faced with adverse conditions. In this case, the resilient attitude will be conditioned by the situation and the context in which the individual operates, when confronting stressful situations in an adaptive way [13]. Other works consider the importance of positive perception and family style that contribute to personality development and learning processes [19]. Thus, the family structure will be determined by the strengths of the system, such as the sum of the different personalities that make up the family nucleus [10]. Family structure and development are essential since they favor the development of resilient attributes, both individually and in the child with a developmental disorder who has transitory or permanent needs, contributing to greater life satisfaction [16].

The relationship between parental stress or adversity caused by the characteristics of children with developmental disorders, as well as resilient attributes or coping capacity are related to life satisfaction, influencing decisively the positive interactions that occur among its members [19]. The factors that are related to well-being and life satisfaction influence family functioning, favoring emotional well-being and allowing a better adaptation to the unexpected circumstances of the child with the developmental disorder [20]. Similarly, when there is less life satisfaction in the family nucleus, an environment of frustration and uncertainty is generated [16].

Stress is a term that is directly related to resilience. Resilience is understood as the adaptive response that a person activates when faced with adverse conditions, as resilience cannot be developed if there are no stressful situations to deal with. These stressful situations and the way in which the person copes with them strengthen families of children with developmental disorders. Different research shows the importance of the family’s attitude towards these stressful situations, which may perceive the disability as a positive experience, functioning as a protective aspect, or negative, as an aspect of resistance [21]. Having a resilient personality does not exempt the person from being able to cope equally well with all adverse situations that they meet. It will also depend on the situation itself, as well as the vital moment in which they find themselves, the context that surrounds them, etc. In other words, having a resilient personality does not mean that stressful situations are never experienced, but they know how to handle them, cope with them, and overcome them [22]. The dynamics of the family are of great importance, as they will favor the development of resilience and contribute towards improving their quality of life [23].

**Figure 1:**
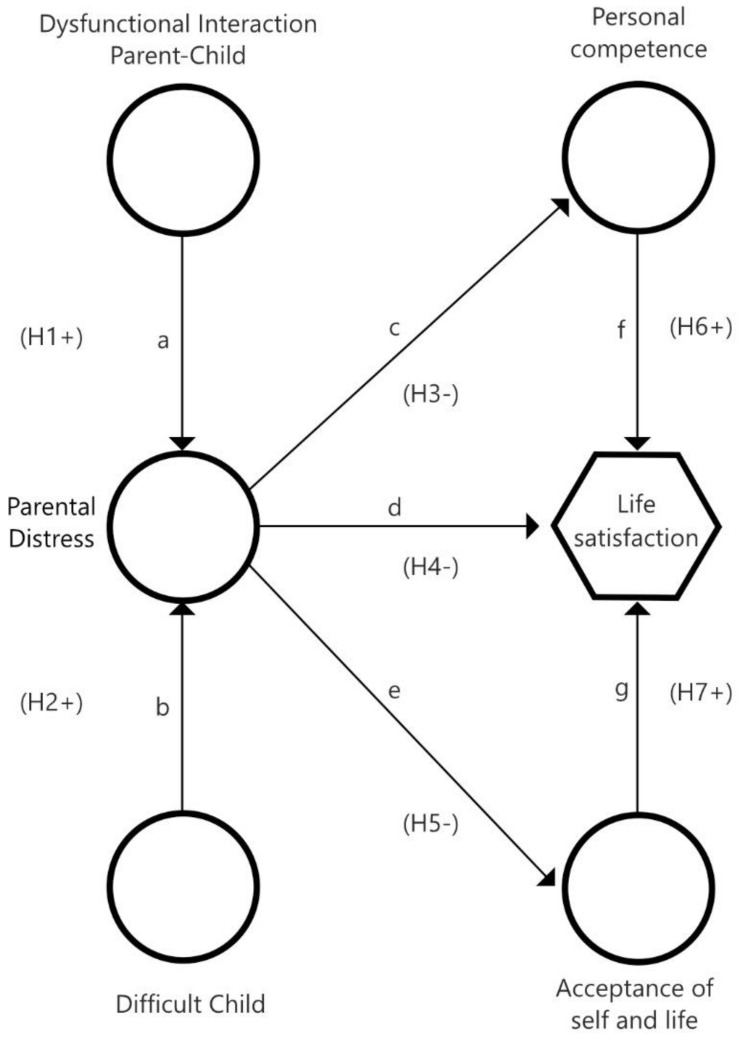
Theorical Model proposed.

Based on the preceding theoretical contributions, the main purpose of this research was to analyze the mediating effect of resilient attributes between parental stress and life satisfaction in families with children with temporary or permanent needs, considering the following hypotheses for its development (Figure 1):

Different research has shown that parental stress and problems related to the child’s development are linked. This influences the degree of one on the other in a reciprocal way [24]. Therefore, it causes an alteration in the behavior of the parents, and in a subsidiary way, reinforces the child’s behavioral problems [25].

However, it has been shown that parents with children with developmental disorders experience a higher level of stress compared to those with children without any disorders [26]. These parents often experience more anxiety in their family life, social dysfunction, and feelings of distress, related to the child’s symptoms [27]. However, in child populations with temporary or permanent needs, there is less research regarding the role of family and parent factors in relation to additional psychopathology. Some studies have reported that parental stress, parental overcontrol style, and domestic chaos are associated with higher proportions of behavior problems, while parental affection and limit setting are related to lower levels of behavioral problems in children with temporary or permanent needs [28].

**Hypothesis 1 (H1).**  *Parent–Child Dysfunctional Interaction and Parental Distress are related*.

**Hypothesis 2 (H2).**  *Child Difficulty and Parental Distress are related*.

Parental stress in parents of children with developmental disorders can manifest itself in various ways. A reduction in self-esteem and life satisfaction in their role as parents has been observed, which means that they feel less secure and satisfied in their ability to raise their children [22]. In the same way, they experience a lower sense of competence and acceptance, which implies that they are less capable of facing and solving the challenges that arise in raising their children. These parents also have lower expectations for success, which means that they do not expect to achieve positive results or achieve high goals in relation to their children’s development and behavior. This reduction in expectations and emotional burden may be due to the additional difficulties faced by daily demands [21].

**Hypothesis 3 (H3).**  *Parental Distress will be negatively related to Personal competence*.

**Hypothesis 4 (H4).**  *Parental Distress will be negatively related to Life satisfaction*.

**Hypothesis 5 (H5).**  *Parental Distress will be negatively related to Acceptance of self and life*.

Parental stress and resilience variables, such as personal competence and acceptance of self and life, are closely related to life satisfaction. Their attitude towards these situations is essential as a protective factor or as a negative resistance. The positive or negative perceptions, in conjunction with a parental organizational style, are important aspects of coping with stress. However, the attitude or the confrontation will be determined by the family structure. Family characteristics include satisfactory relationship patterns, expressions of affection, and respect among members, allowing better adaptability to adverse situations. Similarly, family satisfaction influences the functioning and perception of their role as parents, favoring emotional well-being and better coping with unexpected situations [23]. Communication, family resources, and stress are other elements that are related to satisfaction and influence, to some extent, on their well-being. Higher family satisfaction affects both family functioning and parents’ perception of their new parental role. It promotes emotional well-being and enables more effective adaptation to disability [24]. The acquisition of parenting skills is a complex process that is influenced by a number of variables, such as the innate abilities of each individual, the learning processes, and the positive or negative parenting experiences at earlier stages of their lives. However, in assessing parental satisfaction, it is important to pay attention to the sense of competence. This term refers to how men and women perceive and experience their role as parents. Although there is no consensus on the specific components that constitute it, some more inclusive proposals with children with temporary or permanent needs highlight the following aspects as parental satisfaction or positive perceptions of parenting outcomes in comparison to initial expectations and stresses generated [29].

**Hypothesis 6 (H6).**  *The moderating effect of Personal Competence will be determined by greater life satisfaction*.

**Hypothesis 7 (H7).**  *The moderating effect of Acceptance of self and life will determine a greater life satisfaction*.

## 2. Materials and Methods

### 2.1. Participants

The target population of our research is parents of boys and girls from 0 to 6 years old with developmental disorders who attend Early Childhood Care Centers (CAIT) of the Province of Jaén (Andalusia) [Spain]. According to the General Secretariat for the Family of the Department of Health and Family, there are a total of 1073 (Table 1). The participants (*n* = 123) were fathers and mothers who agreed to participate in the development of the research, verifying the adequacy of the number of relationships established between the selected variables (5), being the statistical power 121 to correctly estimate the model with a significance of 95%. Subsequently, multivariate outliers were examined by calculating and evaluating the Mahalanobis distance squared [24]. Family members were collected individually when attending the CAIT AINPER-LINARES. It was explained that participation in the study was voluntary and they were informed that the data would be treated anonymously. Family members who agreed to participate in the study signed a consent form and then the evaluator explained the instructions of the questionnaires, clarifying any doubts about their completion.

### 2.2. Instruments

The Abidin scale *Parenting Stress Index- Short Form (PSI-SF)* is used to assess parental stress. It consists of 36 items distributed in three dimensions. Dysfunctional parent–child interactions: (PD; e.g., “I feel trapped by my responsibilities as a parent”, “I feel lonely and friendless”), assesses the degree to which the mother or father believes that his child does not meet his expectations and his interactions are not satisfying; child’s difficulties: (PD; e.g., “Sometimes I feel that my child doesn’t appreciate to me and doesn’t want to be near me”), assesses the perception that the mother or father presents towards their child’s behavior (easy or difficult) and vital stress: (PD; e.g., “My son/daughter demands more than the most children”), measures the tensions that mothers or fathers experience in their role as parents. It is evaluated using a Likert-type scale from 1 (totally agree) to 7 (totally disagree). It has a Cronbach’s alpha reliability of 0.91 (total stress). Díaz-Herrero et al. [26] indicate that the total reliability (total stress) of Cronbach’s Alpha is 0.91, similar to those found in other investigations with the same theme.

The Spanish version of the *Resilience Scale (RS-14)* [26] was designed by Wagnild [27]. It measures the degree of resilience, considered as a positive personality characteristic that allows the adaptation of the individual to adverse situations. The RS-14 measures two dimensions: personal competence (11 items, self-confidence, independence, decision, resourcefulness, and perseverance) and acceptance of self and life (3 items, adaptability, balance, flexibility, and a stable outlook on life).

*Satisfaction with Life Scale.* To assess life satisfaction, *the Satisfaction with Life Scale -SWLS-* [30] was used, specifically the version of the Satisfaction with Life Scale by Vázquez et al. [31]. It is composed of five items where participants must indicate the degree of agreement or disagreement for each of the response options of the instrument. The scale in the Spanish version reports an internal consistency of α = 0.82.

### 2.3. Procedure

The ethical guidelines promoted and driven by national and international regulations for conducting research with people were followed. All data were processed in accordance with EU Regulation 2016/679 of the European Parliament and of the Council, of 27 April 2016, both on Personal Data and Organic Law 3/2018, of 5 December, regarding the guarantee of digital rights. Participants were assured that their responses would be kept anonymous and confidential, and that all information provided would be used only for scientific purposes. The instrument was administered individually through the Google^®^ platform (Google – Chrome Web Store). The researchers explained to the participants the purpose of the research, as well as the guidelines for its proper compliance, requesting their voluntary collaboration. The data were collected and its quality was checked, ensuring at all times that the process complied with the ethical principles for research defined in the Helsinki Declaration [32].

### 2.4. Data Analysis

Descriptive statistics (means and standard deviations) were obtained. Previously, the Hot-Deck multiple-entry method was applied to reduce bias by preserving joint and marginal distributions [33,34]. In order to verify the psychometric properties of the questionnaire and obtain the factor loadings of each ítem, the priori validity, reliability (Cronbach’s alpha and Omega coefficient), and consistency of each instrument were analyzed through a Confirmatory Factor Analysis (CFA). The normality analysis was performed by contrasting the multivariate hypothesis, resulting in a non-normal distribution. The analyses were carried out using the SPPS AMOS 25 program, the jamovi software in Version 1.2, and SmartPLS (version 3.3.6). Regarding the coefficients considered in this research, they were the *χ*^2^/*df* ratio, the root mean square error of approximation (RMSEA), the comparative fit index (CFI), and the Tucker–Lewis index (TLI). The goodness of fit of the model was considered satisfactory when the TLI and CFI were ≥ 0.95, and the RMSEA was close to 0.07 [35]. We have used the Partial Least Squares (PLS) technique with an explanatory and predictive purpose of the dependent variables and types of relationships, direct and indirect [36]. Statistical significance required a 95% confidence level (significance *p* < 0.05).

## 3. Results

The assumptions of multicollinearity, homogeneity, and homoscedasticity were analyzed to verify that the resulting distribution met the criteria of dependency between variables. Based on the data obtained with each of the instruments (Table 2), a Confirmatory Factor Analysis (CFA) was performed to verify the validity and internal structure of each item.

The factor loadings for the items of the *Parenting Stress Index-Short Form (PSI-SF)* scale presented an adequate adjustment [37], *χ*^2^/*df* = 1.916, with CFI = 0.913, SRMR = 0.073, and RMSEA = 0.073. The reliability of this scale was Cronbach’s α = 0.922 and McDonald’s ω = 0.924.

The factor loadings for the items of the *Resilience scale (RS-14)* presented an adequate adjustment [36]; *χ*^2^/*df* = 2.183; with CFI = 0.948; SRMR = 0.055; and RMSEA = 0.077. The reliability of this scale was Cronbach’s α = 0.886 and McDonald’s ω = 0.878.

The factor loadings for the *Satisfaction with Life Scale (SWLS)* items presented a moderate adjustment [36]; *χ*^2^/*df* = 1.918; with CFI = 0.898; SRMR = 0.067; and RMSEA = 0.081. The reliability of this scale was Cronbach’s α = 0.714 and McDonald’s ω = 0.748.

### Structural Model

**Figure 2:**
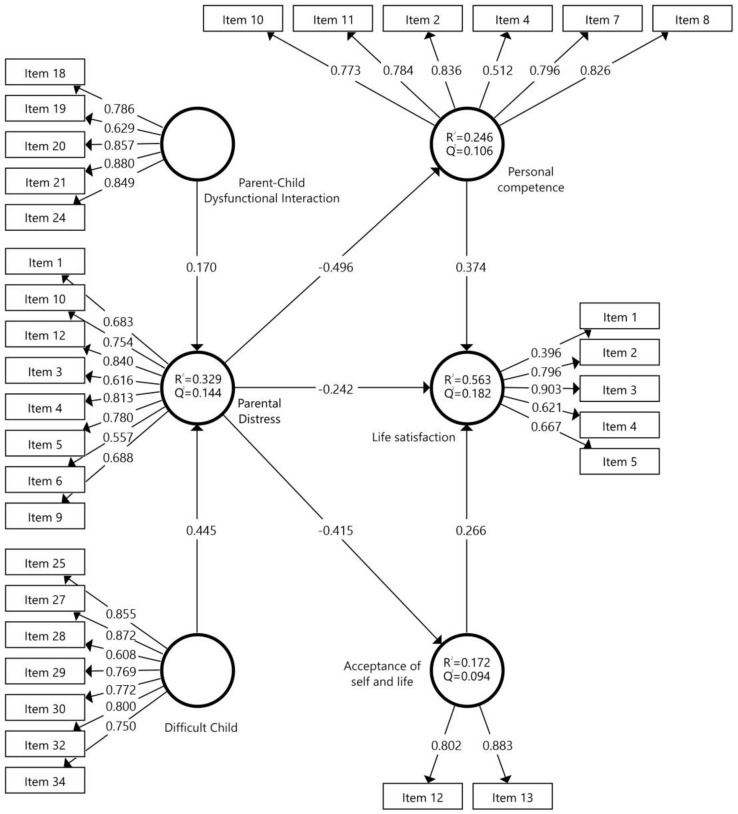
Reliability and validity of the model.

To evaluate the robustness of the factor loadings and the significance between the variables, the Bootstrapping procedure was used with 2000 subsamples [36,37], resulting in the structural model (Figure 2), which reports on the variables considered in this studio. The predictive relevance and standardized regression coefficient or life satisfaction path coefficient [(Q^2^ = 0.182); (*R*^2^ = 0.563)]; parental distress [(Q^2^ = 0.144); (*R*^2^ = 0.329)]; personal competence [(Q^2^ = 0.106); (*R*^2^ = 0.246)]; and acceptance of self and life [(Q^2^ = 0.094); (*R*^2^ = 0.172)], in the estimation of the measurement model, indicated a moderate fit of the model. In this sense, values of R^2^ above 0.67 indicate a substantial adjustment of the model, and above 0.33 a moderate adjustment.

Table 3 shows Cronbach’s alpha, external loads, and the grades of the Composite Reliability Index (CRI). In relation to the convergent validity or degree of certainty, the proposed indicators measure the same latent variable or factor, through the estimation of the average variance extracted (AVE), the values must be greater than 0.5, according to the criteria of Becker et al. [38]. That is, a high value of (AVE) will have a better representation of the load of the observable variable, being metrics used to assess the quality of the structural equation model and the validity of the measurements in a structural equation modeling (SEM) analysis.

The discriminant validity (Table 4) shows the difference between the latent variables, in order to determine the statistical differentiation of each factor with respect to the others, indicating in bold the square root of the extracted mean variance [39].

The discriminant validity (Table 5) was analyzed through the analysis of the cross-loads of each of the latent variables and their respective observed variables, being the loads higher than the rest of the variables [39]. The discriminant validity determines whether a latent variable measures unique concepts and does not overlap too much with other latent variables in the model.

For its part, Table 6 shows the results of the hypothesis contrast, following the criteria of Hair et al. [37], to understand how strong the relationship is between variables in a regression model, controlling the effect of other variables in the model, where the causal relationship with the latent variables can be observed. The *t*-test was obtained (values greater than 1.96 indicate the coherence of the reflective model). In this investigation, the results that showed a higher value were: Parental Distress -> Personal competence (*β* = −0.496, *t* = 4.398, *p* < 0.001); Parental Distress -> Acceptance of self and life (*β* = −0.415, *t* = 2.482, *p* < 0.001) negatively; Child Difficult -> Parental Distress (*β* = 0.445, *t* = 2.366, *p* < 0.001); and Personal competence -> Life satisfaction (*β* = 0.374, *t* = 1.675, *p* < 0.001) positively.

## 4. Discussion

The purpose of this research was to study the mediating effect of resilient attributes between parental stress and life satisfaction in parents with children who present transitory or permanent needs.

In general lines, a significant association has been observed between resilience attributes, such as personal competence and self- and life-acceptance, and the level of stress experienced by parents who have children with temporary or permanent needs [40]. This relationship is characterized by a strong inverse correlation, indicating that as levels of resilience and self-acceptance increase, parents’ perceived stress tends to decrease. In addition, a positive relationship has been identified between the variables of self- and life-acceptance and parental life satisfaction [41]. This finding suggests that the ability to accept oneself and one’s life circumstances may act as a protective or buffering factor against parental stress, contributing to greater life satisfaction in general [42]. These findings are consistent with previous literature highlighting the importance of resilience and self-acceptance as key psychological resources for coping with stressful and adverse situations. They also support the idea that strengthening these competencies may be beneficial not only for parents’ individual well-being but also for the quality of parenting and family adjustment in contexts of children with special needs [40].

Based on the results obtained, it is perceived that resilient attributes have a mediating effect on parental stress and life satisfaction. In this way, and more specifically, this study determines that there is a positive correlation between the binomial *Parental–child dysfunctional interaction* and *Parental Distress (H1)*, confirming that the feeling of competence that parents have not only about themselves but also in the care of their children determines the way in which they deal with certain situations that cause them stress [23]. These data are in line with those results in previous research [25] that determine that the feeling of parental competence affects in the same way the ability to assume the responsibilities that this new role implies, where a great influence of three main dimensions prevails: the personal characteristics of the father or the mother, the personal characteristics of the child as well as the characteristics that predominate between the father/mother and the child [25,43]. It can be argued that working with families can improve the resilience and stress-coping of parents with children with temporary or permanent needs [44].

In the same way, the data obtained have allowed us to verify that there is a positive correlation between *parental stress* and the *difficulty associated with the child (H2).* Coinciding with the contributions of Sánchez et al. [41], stressful situations can be aggravated when the child has a disability since the care and upbringing of the child are influenced not only by not meeting expectations but also by feelings of disappointment, anger, guilt, anguish, and fear, among others. However, these situations usually disappear gradually when the family activates the internal and external resources that are necessary to deal with these situations [45,46].

The results found have also shown that there is a negative correlation between the *Parental Distress variable* and *the Personal competence, Life satisfaction, and Acceptance of self and life* variables *(H3, H4, H5).* These results coincide with previous investigations that determine that the fact of having a resilient personality does not exempt the person from being able to equally face all the adverse situations that they encounter. Therefore, having a resilient personality will depend on the situation itself, the vital moment or the context in which the person finds himself, as well as the tools that he has to deal with certain stressful situations [47]. Resilience in the family environment is made up of both the strength of the family itself as a whole and the sum of the personalities of the different members that make up the family nucleus. Therefore, as Bravo and López [22] affirm, the dynamics that the family has will be of great importance since it will favor the development not only of the individual resilience of each member but also that of the child with disabilities, thus contributing to an improvement in their quality of life and greater adaptability to disability. The relationship between stress and satisfaction is confirmed, although to a lesser extent; in this sense, these results are in line with the few studies that exist in this regard, which identify different aspects that can influence family satisfaction, where stress is one of them [48].

Finally, it has been noticed that resilient attributes are significantly associated with greater life satisfaction *(H6, H7).* A positive correlation is observed between both constructs, determining that the higher the level of resilience, the greater satisfaction with life, being these results conclusive with other studies that follow this same trend [20,49]. Greater life satisfaction will affect both the perception that parents have about themselves as parents, as well as family functioning. This will allow a better adaptation to the disability and, consequently, a greater emotional well-being [16]. On the other hand, when there is less family satisfaction, an environment is generated where sadness, frustration, and depression prevail and, therefore, the presence of high levels of stress lead to less emotional well-being [50].

## 5. Conclusions

Despite the findings obtained, latent limitations in this study when interpreting the results for future work were found, such as the sample size. This may not be representative in comparison with the totality of families with children with permanent or transitory needs. Although this aspect should be taken with caution, it can also serve as a precedent for future studies, extending its projection with larger and more representative samples, and comparison between different centers and longitudinal studies, considering that the acquisition of resilient attributes is beneficial on parental stress and the vital satisfaction of families that care children in vulnerable situations. A set of strategies aimed at family learning and, in particular, at the promotion of parenting skills to promote developmental opportunities for children with developmental disorders, is known in the literature as parent coaching, highlighting its relevance in early intervention [49].

These practices transform the traditional role of the professional, who, through collaboration with families in natural environments, moves from being just an expert to becoming an agent of change [48,50]. From this philosophy, the early intervention professional becomes a promoter of family learning, in charge of promoting effective practices and consolidating them. This practice is based on an agreement between the coach, the early intervention professional, and the family, through which learning opportunities are planned. In order to develop new skills and strategies, the early childhood professional will rely on the observation of the child’s home environment [49]. Understanding the natural environment of the child with a developmental disorder who has temporary or permanent needs will allow reflection on actions that require modifications to reach the set goals and design learning opportunities.

In any case, the veracity and relevance of each of the evidence described will imply a greater knowledge of the constructs analyzed and will serve to improve the implementation of future intervention programs.

## Figures and Tables

**Table 1:** Descriptive analysis.

Gender (Minor)	Frequency	%	Age (M)	SD
Boys	51	41.5%	4.32	(±1.312)
Girls	72	58.5%		
Social Communication (DSM-5-TR)	Frequency	%		
Need support	67	48.8%		
Need noticeable support	28	17.1%		
Need significant support	28	17.1%		
Restricted behavior (DSM-5-TR)	Frequency	%		
Need support	73	53.7%		
Need noticeable support	25	14.6%		
Need significant support	25	14.6%		
Gender (Family)	Frequency	%	Age (M)	SD
Mothers	96	78.0%	37.85	(±5.043)
Fathers	27	22.0%		

Notes: M: Mean. SD: Standard deviation.

**Table 2:** Factor loadings (Composite, Mode A).

Latent Factor	Indicator	α	ω	Estimator	*SE*	*Z*	*p*	*β*	*AVE*	*CR*
Parental Distress	Item 1	0.918	0.921	1.108	0.262	4.22	<0.001	0.620	0.495	0.868
	Item 3	0.921	0.923	1.164	0.292	3.98	<0.001	0.593		
	Item 4	0.917	0.919	1.829	0.277	6.60	<0.001	0.865		
	Item 5	0.917	0.920	1.654	0.263	6.30	<0.001	0.842		
	Item 6	0.920	0.922	1.060	0.312	3.39	<0.001	0.518		
	Item 9	0.921	0.923	1.198	0.318	3.77	<0.001	0.571		
	Item 10	0.919	0.922	1.286	0.331	3.89	<0.001	0.610		
Parent-Child Dysfunctional Interaction	Item 12	0.917	0.920	1.501	0.307	4.89	<0.001	0.717	0.570	0.867
	Item 18	0.919	0.921	1.480	0.269	5.51	<0.001	0.760		
	Item 19	0.922	0.924	1.051	0.285	3.69	<0.001	0.561		
	Item 20	0.917	0.920	1.588	0.279	5.70	<0.001	0.780		
	Item 21	0.915	0.917	1.509	0.245	6.15	<0.001	0.821		
	Item 24	0.916	0.918	1.503	0.244	6.17	<0.001	0.823		
Child Difficult	Item 25	0.918	0.920	1.454	0.236	6.16	<0.001	0.819	0.552	0.894
	Item 27	0.915	0.917	1.623	0.251	6.45	<0.001	0.840		
	Item 28	0.920	0.923	0.860	0.255	3.37	<0.001	0.512		
	Item 29	0.917	0.919	1.547	0.283	5.47	<0.001	0.751		
	Item 30	0.918	0.920	1.382	0.263	5.26	<0.001	0.733		
	Item 32	0.914	0.916	1.453	0.253	5.75	<0.001	0.780		
	Item 34	0.916	0.918	1.404	0.276	5.08	<0.001	0.715		
Personal competence	Item 2	0.859	0.867	0.899	0.166	5.41	<0.001	0.749	0.598	0.853
	Item 4	0.878	0.889	0.757	0.224	3.38	<0.001	0.517		
	Item 7	0.859	0.869	1.063	0.195	5.46	<0.001	0.752		
	Item 8	0.846	0.858	1.667	0.251	6.64	<0.001	0.861		
	Item 10	0.867	0.876	0.738	0.183	4.04	<0.001	0.611		
	Item 11	0.859	0.869	0.930	0.194	4.79	<0.001	0.691		
Acceptance of self and life	Item 12	0.872	0.880	1.058	0.286	3.70	<0.001	0.570	0.479	0.811
	Item 13	0.856	0.867	1.056	0.215	4.92	<0.001	0.751		
Life satisfaction	Item 1	0.743	0.770	0.600	0.237	2.53	<0.001	0.379	0.508	0.852
	Item 2	0.640	0.694	0.951	0.233	4.09	<0.001	0.627		
	Item 3	0.536	0.567	1.620	0.233	6.96	<0.001	0.907		
	Item 4	0.700	0.738	0.885	0.294	3.01	<0.001	0.469		
	Item 5	0.678	0.726	0.658	0.196	3.35	<0.001	0.517		

*Notes: CR: Composite reliability. AVE: Average variance extracted. Significant at p < 0.05 (2 tails).*

**Table 3:** Correlation Weights, Reliability Estimates, and Convergent Validity Statistics.

Variable	*α*	Composite Reliability (CR)	Rho_A	Average Variance Extracted (AVE)
Parental Distress	0.867	0.896	0.889	0.522
Parent-Child Dysfunctional Interaction	0.867	0.901	0.935	0.649
Child Difficult	0.890	0.915	0.909	0.607
Personal competence	0.852	0.891	0.878	0.581
Acceptance of self and life	0.700	0.831	0.723	0.712
Life satisfaction	0.721	0.817	0.802	0.587

Note: The one-tailed 95% percentile confidence intervals [5%, 95%] of the reliability and validity statistics have been provided. CR = composite reliability; AVE = Average Variance Extracted.

**Table 4:** Measurement model. Discriminant validity.

Fornell–Larcker Criterion	1	2	3	4	5	6
Parental Distress	**0.723**					
2. Parent-Child Dysfunctional Interaction	0.470	**0.805**				
3. Child Difficult	0.560	0.672	**0.779**			
4. Personal competence	−0.496	−0.214	−0.287	**0.762**		
5. Acceptance of self and life	−0.415	−0.075	−0.215	0.761	**0.844**	
6. Life satisfaction	−0.537	−0.341	−0.424	0.696	0.651	**0.698**
**Heterotrait–Monotrait ratio (HTMT)**	**1**	**2**	**3**	**4**	**5**	**6**
Parental Distress						
2. Parent-Child Dysfunctional Interaction	0.523					
3. Child Difficult	0.618	0.745				
4. Personal competence	0.546	0.250	0.336			
5. Acceptance of self and life	0.527	0.170	0.349	0.889		
6. Life satisfaction	0.722	0.445	0.499	0.815	0.867	

Note: Fornell–Larcker criterion: Diagonal elements (bold) are the square root of the variance shared between the constructs and their measures (average variance extracted). Off-diagonal elements are the correlations between constructs. For discriminant validity, diagonal elements should be larger than of-diagonal elements.

**Table 5:** Cross loads (latent and observable variables).

Variable	Parental Distress	Parent-Child Dysfunctional Interaction	Child Difficult	Personal Competence	Acceptance of Self and Life	Life Satisfaction
Parental Distress						
Item 1	0.683	0.391	0.435	−0.503	−0.431	−0.296
Item 10	0.754	0.381	0.357	−0.356	−0.231	−0.414
Item 12	0.840	0.418	0.460	−0.534	−0.387	−0.632
Item 4	0.813	0.358	0.476	−0.310	−0.269	−0.399
Item 5	0.780	0.326	0.461	−0.190	−0.149	−0.258
Item 6	0.557	0.473	0.350	−0.078	−0.138	−0.222
Item 9	0.688	0.273	0.278	−0.445	−0.397	−0.390
Parent-Child Dysfunctional Interaction						
Item 18	0.229	0.786	0.532	−0.037	0.130	−0.204
Item 19	0.189	0.629	0.300	−0.101	−0.012	0.075
Item 20	0.385	0.857	0.503	−0.133	−0.047	−0.210
Item 21	0.524	0.880	0.600	−0.270	−0.112	−0.493
Item 24	0.402	0.849	0.685	−0.214	−0.146	−0.289
Child Difficult						
Item 25	0.342	0.403	0.855	−0.268	−0.208	−0.337
Item 27	0.566	0.507	0.872	−0.208	−0.177	−0.367
Item 28	0.389	0.291	0.608	−0.328	−0.338	−0.341
Item 29	0.331	0.652	0.769	−0.077	−0.077	−0.257
Item 32	0.533	0.637	0.800	−0.198	−0.141	−0.378
Item 34	0.405	0.704	0.750	−0.358	−0.264	−0.389
Personal competence						
Item 11	0.247	0.033	0.093	0.784	0.619	0.552
Item 2	0.445	−0.185	−0.269	0.836	0.549	0.555
Item 4	−0.200	−0.070	−0.176	0.512	0.480	0.255
Item 7	−0.429	−0.219	−0.225	0.796	0.613	0.505
Item 8	−0.524	−0.324	−0.324	0.826	0.758	0.572
Acceptance of self and life						
Item 12	−0.249	−0.079	−0.259	0.570	0.802	0.514
Ítem 13	−0.433	−0.052	−0.121	0.704	0.883	0.581
Life satisfaction						
Item 1	−0.559	−0.271	−0.423	0.016	0.093	0.396
Item 2	−0.413	−0.167	−0.130	0.610	0.617	0.796
Item 3	−0.508	−0.334	−0.395	0.636	0.594	0.903
Item 4	−0.373	−0.288	−0.439	0.431	0.243	0.621
Item 5	−0.150	−0.201	−0.278	0.497	0.508	0.667

**Table 6:** Coeficiente path (standardized regression coefficient).

Relation between Variables	Route Coefficient (*β*)	Standard Deviation (*σ*)	Statistical *t*	*p*
Parental Distress -> Personal competence	−0.496	0.113	4.398	***
Parental Distress -> Acceptance of self and life	−0.415	0.167	2.482	***
Parental Distress -> Life satisfaction	−0.242	0.207	1.168	0.243
Parent-Child Dysfunctional Interaction -> Parental Distress	0.170	0.182	0.935	0.350
Child Difficult -> Parental Distress	0.445	0.188	2.366	***
Personal competence -> Life satisfaction	0.374	0.223	1.675	***
Acceptance of self and life -> Life satisfaction	0.266	0.169	1.572	0.117

Note: *** = *p* < 0.001.

## Data Availability

The datasets generated during and/or analyzed during the current study are available from the corresponding authors upon reasonable request.
